# Urbanization Impacts on Mammals across Urban-Forest Edges and a Predictive Model of Edge Effects

**DOI:** 10.1371/journal.pone.0097036

**Published:** 2014-05-08

**Authors:** Nélida R. Villaseñor, Don A. Driscoll, Martín A. H. Escobar, Philip Gibbons, David B. Lindenmayer

**Affiliations:** 1 ARC Centre of Excellence for Environmental Decisions, the NERP Environmental Decisions Hub, The Fenner School of Environment and Society, The Australian National University, Canberra, ACT, Australia; 2 School of Natural Resources, Universidad de Chile, Santiago, Chile; Institute of Agronomy, University of Lisbon, Portugal

## Abstract

With accelerating rates of urbanization worldwide, a better understanding of ecological processes at the wildland-urban interface is critical to conserve biodiversity. We explored the effects of high and low-density housing developments on forest-dwelling mammals. Based on habitat characteristics, we expected a gradual decline in species abundance across forest-urban edges and an increased decline rate in higher contrast edges. We surveyed arboreal mammals in sites of high and low housing density along 600 m transects that spanned urban areas and areas turn on adjacent native forest. We also surveyed forest controls to test whether edge effects extended beyond our edge transects. We fitted models describing richness, total abundance and individual species abundance. Low-density housing developments provided suitable habitat for most arboreal mammals. In contrast, high-density housing developments had lower species richness, total abundance and individual species abundance, but supported the highest abundances of an urban adapter (*Trichosurus vulpecula*). We did not find the predicted gradual decline in species abundance. Of four species analysed, three exhibited no response to the proximity of urban boundaries, but spilled over into adjacent urban habitat to differing extents. One species (*Petaurus australis*) had an extended negative response to urban boundaries, suggesting that urban development has impacts beyond 300 m into adjacent forest. Our empirical work demonstrates that high-density housing developments have negative effects on both community and species level responses, except for one urban adapter. We developed a new predictive model of edge effects based on our results and the literature. To predict animal responses across edges, our framework integrates for first time: (1) habitat quality/preference, (2) species response with the proximity to the adjacent habitat, and (3) spillover extent/sensitivity to adjacent habitat boundaries. This framework will allow scientists, managers and planners better understand and predict both species responses across edges and impacts of development in mosaic landscapes.

## Introduction

Urbanization is a strong driver of environmental modification worldwide [1], [2]. Currently, there are more than seven billion people on earth, with more than half living in urban areas [3]. By 2050, more than 70% of the human population will live in urban areas [4]. Therefore, the pressure for urban development will lead to continued urban expansion. These changing environmental conditions will cause loss, degradation, fragmentation and isolation of remnant habitats [5]; and affect biodiversity at local, landscape and regional scales [6], [7].

As a result of urbanization, animals are increasingly exposed to urban boundaries with different edge contrasts [8], [9]. Edge contrast, defined as the difference in composition or structure between adjoining ecosystems at both sides of the boundary [10], is a key element influencing the movement of animals across landscapes [11]. Indeed, metapopulation persistence relies on emigration, colonization and isolation [12], all of them influenced by how animals move and distribute in relation to proximity of habitat boundaries (i.e. “edge effects”) [13]. It is expected that a boundary with high contrast between juxtaposed patches (i.e. a “hard edge”) will generate a more pronounced reduction in the movement of animals than a “softer edge” [8], [14]–[16], leading to a differential length, depth or penetration of edge effects [10].

Although edge effects have received extensive attention in the literature, most knowledge on edge effects comes from forested patches adjacent to pastures or crops [8], [10], [11], [16], [17]. Little is known about the edge effects caused by different kinds of urban development. Given the rapid and accelerating expansion of urban areas, the lack of attention to biodiversity in the wildland-urban interface is a major knowledge gap [18]. Further, the study of ecological processes along habitat edges has been restricted to a focal patch (i.e. “one side” of the edge) (e.g. [10], [19]) and on a small spatial scale (but see [20], [21]), limiting our understanding of how species respond to contrasting edges to effectively guide management and urban planning.

**Figure 1:**
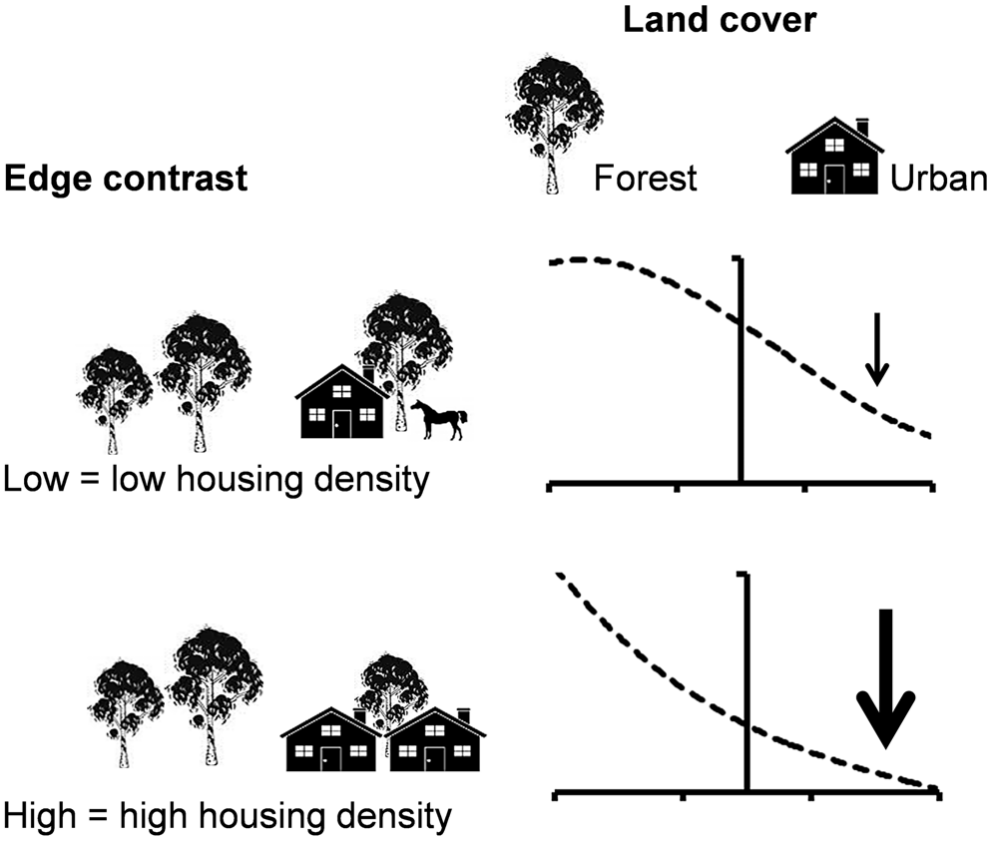
Expected responses of arboreal marsupials according to edge contrast and land cover in south-eastern Australia. Graphs show the predicted trajectory of the animal abundance (dashed line) in adjacent habitats. The vertical line in each graph represents the boundary between two habitat patches. Arrows represent direction and magnitude of the predicted response with increasing distance from the urban boundary by each combination of edge contrast and land cover.

In this study, we explore the response of animals on both sides of urban boundaries at a large spatial scale. We focused on arboreal marsupials because they are sensitive to changes in land cover and landscape fragmentation as a result of their dependence on forest resources (e.g. foliage, tree hollows) [22], [23]. We measured the response of arboreal marsupials in urban-forest edges, and compared different edge contrasts based on the style of urban development (i.e. housing density) adjacent to relatively intact forest. We also surveyed forest controls located away from any type of development to detect whether edge responses extended beyond the edge length defined in our study. Low (i.e. “soft”) and high (i.e. “hard”) contrast edges corresponded to intact forest adjacent to low and high-density housing developments, respectively (Figure 1).

When a patch of habitat (e.g. forest) is juxtaposed with a patch of lower-quality habitat (e.g. an urban area) and the type of resources are qualitatively the same in both patch types (e.g. trees that provide foliage and den sites), a gradual change in species abundance from the highest densities in the interior of the higher quality habitat to the lower densities in the interior of the lower quality habitat is expected across the edge [17], [24] (Figure 1). Because soft edges typically produce a weaker response among biota than hard edges [10] (Figure 1), at the outset of this investigation, we predicted a transitional response characterized by: (a) a reduction of arboreal marsupial abundance in urban areas with a reduced magnitude of the effect in soft edges; (b) a longer spillover of arboreal marsupials from forest into urban areas with soft edges; and (c) a longer penetration of the negative effect on arboreal marsupials of urban areas into forests with hard edges. Our results have relevance for guiding both planning and management strategies to improving the conservation of forest-dwelling animals in urban landscapes, particularly those at the wildland-urban interface.

## Materials and Methods

### Ethics Statement

Our study was observational and no plants or animals were harmed. The project was conducted in accordance with the requirements of permit A2012/52 issued by the Animal Experimentation Ethics Committee of The Australian National University. We also obtained a Permit for an Activity in a Commonwealth Reserve (BDR12/00010), a scientific research license issued by the New South Wales National Parks and Wildlife Service (SL101012) and a Special Purposes Permit for Research in Forests NSW (SPPR0010) granted to NRV. No specific permits were required for surveying public tracks or private lands, where residents and land owners approved access.

### Study Area

**Figure 2:**
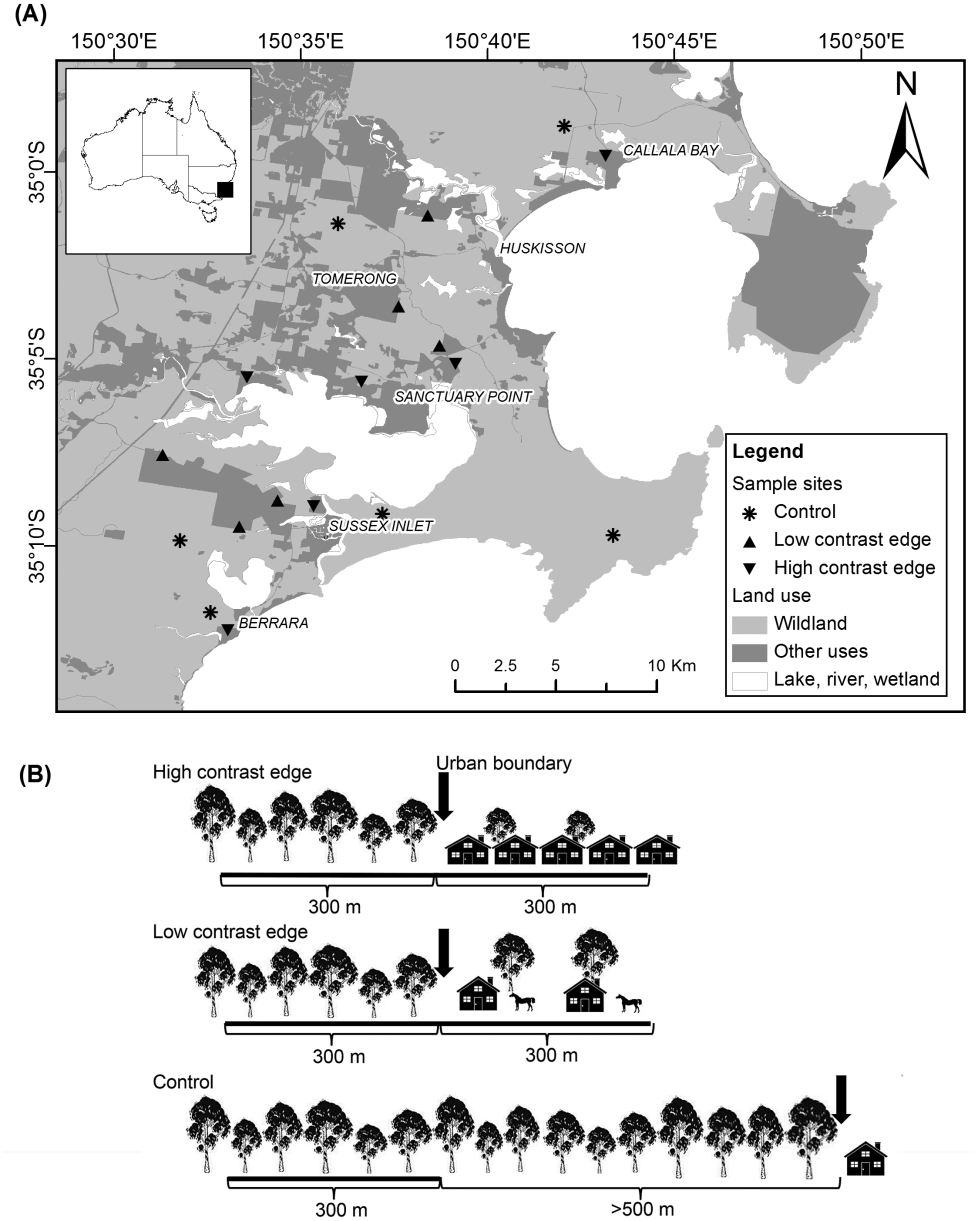
Study area, sites and diagram of transect placement for spotlighting surveys in south-eastern Australia. (A) Study area and sites of spotlight surveys in south-eastern Australia. (B) Diagram of the transect placement for spotlighting surveys in each edge contrast. At each of the high and low contrast edges a 300 m transect was established from the urban boundary into the forest and another 300 m transect was established from the urban boundary into the urban area. Control sites included a 300 m transect within a forest and were >500 m away from other land use. Arrows indicate the urban boundary.

Our study area was located on the south coast of New South Wales, south-eastern Australia. It encompasses an area between the towns of Callala Bay (34°59′S 150°43′E) and Berrara (35°12′S 150°33′E), and covers approximately 500 km^2^ (Figure 2A). The region has a temperate climate, with warm summers and cold winters. Annual mean minimum and maximum air temperatures are 13.8°C and 20°C, respectively. Annual rainfall is ca. 1,000 mm and spread evenly throughout the year [25].

The area we studied is heavily dominated by native eucalypt forests. Natural lands cover 81.4% of the landscape, followed by urban areas (13.4%) and a small percentage of other land uses (e.g. grazing, cropping, mining; 5.3%) [26]. We selected this area dominated by eucalypt forests to reduce landscape-scale variation across sites. Currently, high human population growth and an increasing demand for holiday houses along the coast are triggering further clearing of vegetation for urban development. This land use change is creating urban areas of different housing densities interspersed with natural areas such as national parks and reserves.

### Site Selection

To study the effect on arboreal marsupials of edge contrast (i.e. housing density), land cover (i.e. forest and urban) and distance to the urban boundary across urban-forest edges, we compiled detailed surveys at 12 treatment sites (i.e. forest-urban edges) and six control sites. To select treatment sites, we first identified urban cover with high and low housing densities in a land use shapefile [26] in ArcMap 10 (ESRI). High-density housing developments were represented by residential zones dominated by single storey houses (average block size: 0.06 ha). Low-density housing developments corresponded to rural residential zones with allotments from 0.2 to 16 ha in size. We identified potential sites in urban boundaries adjacent to large areas of forest (i.e. forest extending beyond 600 m from the urban boundary and away from other land uses); and selected a subset of six sites randomly in each category of housing density (i.e. high and low). We restricted our sampling to places where: (1) the cover type and housing density were readily assigned to the key design structure in our study, and (2) the forest supported key elements of stand structural complexity for arboreal marsupials (i.e. large trees and cavities) [27]. Finally, six control sites were selected randomly in large forested areas at least 0.5 km away from any other land use, but within 8 km of a treatment site (mean nearest distance from an urban boundary ± se = 1447 m±400 m). Control sites were located within state forests, national parks and reserves and were constrained to sites that contained key elements of stand structural complexity for arboreal marsupials.

At each treatment site, we established a 300 m transect along streets or public tracks from the urban boundary into the urban cover and another 300 m transect was established along an unpaved track from the urban boundary into the forest (Figure 2B). At each control site, a 300 m transect was established on an unpaved track (Figure 2B).

### Surveys of Arboreal Marsupials

To estimate species richness and the abundance of arboreal marsupials, we used line-transect sampling [28]. We spotlighted along each transect in our study sites. This method is widely used in Australia [29], [30] and produces the best results for this group of animals in forests when compared with other survey techniques [31].

Our 18 sites (comprising 30 transects: two transects per treatment site and one transect per control site) were surveyed up to four times (111 passes, mean ± se = 3.4±0.6) between December 2012 and February 2013. We accounted for uneven survey effort in our analysis (see Data Analysis). All transects were surveyed on foot at a speed of ca. 10 m/minute by using a 30-W spotlight (LightFORCE) to detect animals by their eyeshine, body size, and other physical characteristics with help of binoculars. For each detection, we recorded species, the position of the observer along the transect line, the distance between observer and animal, and the perpendicular distance of the animal to the transect line.

All spotlighting surveys started 1 hour after dusk and ended before 03h00. We standardized weather and temporal factors to limit their effects on the abundance index by restricting the surveys to good weather conditions (i.e. we did not perform surveys during medium or strong wind or rain). We also avoided surveying within four days of a full moon due to possible changes in animal activity [32]. To limit observer effects on our data, 75% of transects were surveyed by two observers (MAHE and NRV), each recording at a different time and from a different direction but on the same night.

### Vegetation Surveys

Habitat characteristics in each spotlighting transect were quantified in terms of vegetation structure and composition, by using the point-intercept survey method [33] along a 50 m transect (50 points) at 100 and 300 m from the urban boundary in both directions (i.e. urban area and forest). All vegetation transects were placed on the vegetation parallel to the spotlighting transects (including street vegetation and front gardens in urban areas). At each point, we recorded the presence/absence of grass, litter, bare ground, impervious surfaces, woody debris, understory vegetation (excluding grass) and canopy. The proportion cover of each habitat variable was calculated by dividing the amount present by the total number of points (50) on each transect. We averaged the proportions recorded along the two vegetation transects to characterize each spotlighting transect.

### Data Analysis

#### Species richness, total abundance and individual species abundance

We aggregated our data on all animals recorded in different passes of each transect. For each record, we calculated the distance of the animal to the urban boundary in ArcGIS 10. Each animal was assigned to one of three distance intervals from the urban boundary: 0–100 m, 100–200 m and 200–300 m. Although distance to the urban boundary was not considered in control sites, all records were grouped by 100 m transect to ensure the same sampling unit was used across all analysis (see below). The midpoint of each distance interval was used as a continuous variable (i.e. 50 m, 150 m and 250 m) in later analysis.

Prior to conducting detailed statistical analysis, we ensured that the species’ detection did not differ between urban and forest cover. We compared the distribution function of the proportion of animals seen according to the distance to the observer in forest versus urban cover by using a bootstrapping version of the Kolmogorov-Smirnov test. The Kolmogorov-Smirnov test examines the null hypothesis that samples are drawn under the same distribution [34]. None of the species exhibited a different probability distribution of records between urban and forest transects (*P*>0.2), suggesting a similar rate of detection between land cover types.

We used Generalized Lineal Mixed Models (GLMMs) with Laplace approximation [35] and Poisson link function for analysing the effect of housing density, land cover and distance to the urban boundary on the richness, total abundance and individual species abundance per 100 m spotlight transect in forest-urban edges (12 treatment sites). We selected only those species with ≥15 records to perform species-level analysis. Fixed effects included housing density, land cover, their interaction, and the distance to the urban boundary nested within the interaction of housing density and land cover. The term distance to the urban boundary nested within the interaction of housing density and land cover allowed distance to the urban boundary to have a different effect (e.g. positive, negative or neutral) in each combination of housing density and land cover. The site (i.e. transect location, including the adjacent urban and forest transects) was fitted as a random effect. Then, each response variable was modelled in a GLMM as:

Response ∼ HD+LC+(HD×LC)+(HD×LC/D)+(1| S) (1)

Where:

HD = housing density, factor with two levels: high and low.

LC = land cover, factor with two levels: urban and forest.

D = distance to the urban boundary, continuous scale.

S = site, factor with 12 levels.

As the survey effort was not the same in all transects, we modelled the natural logarithm of the number of passes as an offset variable in all models. We tested for overdispersion in our models by comparing the residual deviance with the residual degrees of freedom. When a model was overdispersed, an observation-level random effect was added to the model (i.e. each statistical unit, 100-m transect, was modelled as a random effect) [36].

Because we allowed distance to the urban boundary to have a different effect in each combination of housing density and land cover (HDxLC/D in equation 1), each GLMM was first tested for the effect of the distance to the urban boundary between each combination of housing density and land cover with Wald *X* ^2^ contrast tests. When there was no significant effect of distance to the urban boundary, a backward elimination procedure was used to remove non-significant variables and select the best models. Wald *X* ^2^ tests were performed to evaluate the significance of a factor in each model [35]. When the interaction between housing density and land cover was significant, multiple comparison tests were performed using Fisher’s LSD. We regarded results as significant when *P*<0.1 to identify all relevant trends.

When the model selection discarded both distance to the urban boundary and land cover as relevant predictors (i.e. HDxLC/D, HDxLC and LC in equation 1), the response was not different across the edge. If there were any edge effects, they extended further than 300 m from the urban boundary. In those circumstances, we tested for such deeper edge effects by incorporating control sites in a new analysis (i.e. including all 18 sites regarding a 100-m transect as the statistical sampling unit). Then, edge contrast was a variable with tree levels: high contrast edge, low contrast edge and forest control. This approach allowed us to test whether edge effects of urban areas with different housing densities extend further than 300 m into the adjacent forest.

Once the backward selection was completed, we estimated the predicted response values from the relevant parameters of the final GLMMs, and estimated standard errors [37]. We evaluated potential spatial autocorrelation in the residuals of our final models, to test whether the assumptions of independence and distribution of residuals were violated [38]. We used residual variograms to visualize whether the residual semivariance (i.e. a measure of the variance of model residuals between sites) was independent of distance between sites. We also calculated Moran’s I index for residuals from our final models. Moran’s I index tests the null hypothesis of no correlation between model residuals given a matrix of distances between sites (1/distance) that is used as a “neighbourhood” weight [39].

#### Vegetation

We described differences in vegetation structure among transect classes (i.e. each combination of edge contrast and land cover, and control sites) to help identify habitat characteristics and interpret observed responses in arboreal marsupials by using Principal Components Analysis (PCA). We used PCA on a correlation matrix of the vegetation variables from the point-intercept method (i.e. proportion of grass, litter, bare ground, impervious surface, woody debris, understory vegetation and canopy cover). We log transformed variables where appropriate and tested for significant differences in the component scores among transect classes with analysis of variance (ANOVA) for the first three components. When significant differences were found, we performed Tukey’s HSD to identify what classes were different.

All statistical analyses were performed in R-2.15.2 [40]. We used the package “Matching” for bootstrapping of Kolmogorov-Smirnov tests [34], “lme4” to fit GLMMs [41], “AICcmodavg” to obtain predicted values and standard errors [37], “gstat” to calculate residual variograms [42] and “ape” for Moran’s I autocorrelation index [39].

## Results

We recorded 164 individuals of six species of arboreal marsupials (Table S1) which represent most species of arboreal marsupials described for the study area. The number of records allowed us to perform species-level analysis for four species: the common brushtail possum (*Trichosurus vulpecula*, Kerr 1792), the common ringtail possum (*Pseudocheirus peregrinus*, Boddaert 1785), the sugar glider (*Petaurus breviceps*, Waterhouse 1839), and the yellow-bellied glider (*Petaurus australis*, Shaw 1791). The greater glider (*Petauroides volans*, Kerr 1792) and the feathertail glider (*Acrobates pygmaeus*, Shaw 1794) were recorded three times and once, respectively. Data for these two species were insufficient to enable species-level analysis, but records for them were used in analyses of total abundance and species richness.

### Total Abundance

**Figure 3:**
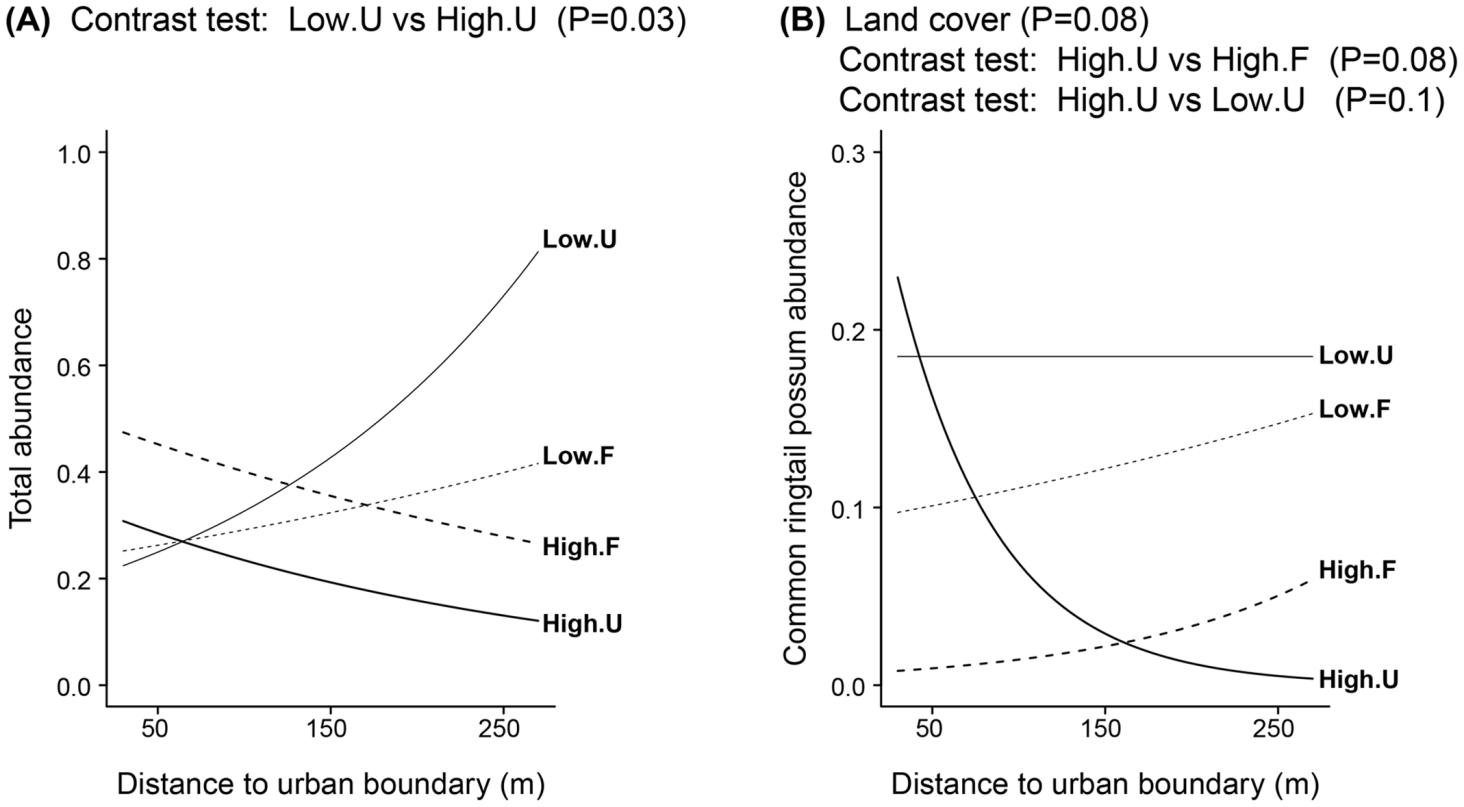
Predicted mean abundances per 100 m spotlight transect according to the distance to the urban boundary from Generalized Linear Mixed Models. (A) Total abundance of arboreal marsupials and (B) common ringtail possum abundance. Codes of edge contrasts: High = high housing density; Low = low housing density. Codes of land cover: F = forest; U = urban. Estimated values were predicted for a single spotlighting pass. Significant *P*-values of the relevant variables in the GLMMs and significant contrast tests are shown on the top of each graph.

The total abundance of arboreal marsupials differed with distance to the urban boundary when we compared urban cover with different housing densities (Figure 3A). In urban cover with high housing density, the abundance of arboreal marsupials declined with distance to the urban boundary; whereas in urban cover with low housing density, the abundance of arboreal marsupials increased with distance to the urban boundary (Wald contrast test, *P* = 0.03) (Figure 3A). Observed trends in forest cover were not significantly different (*P*>0.1).

### Species Richness

**Figure 4:**
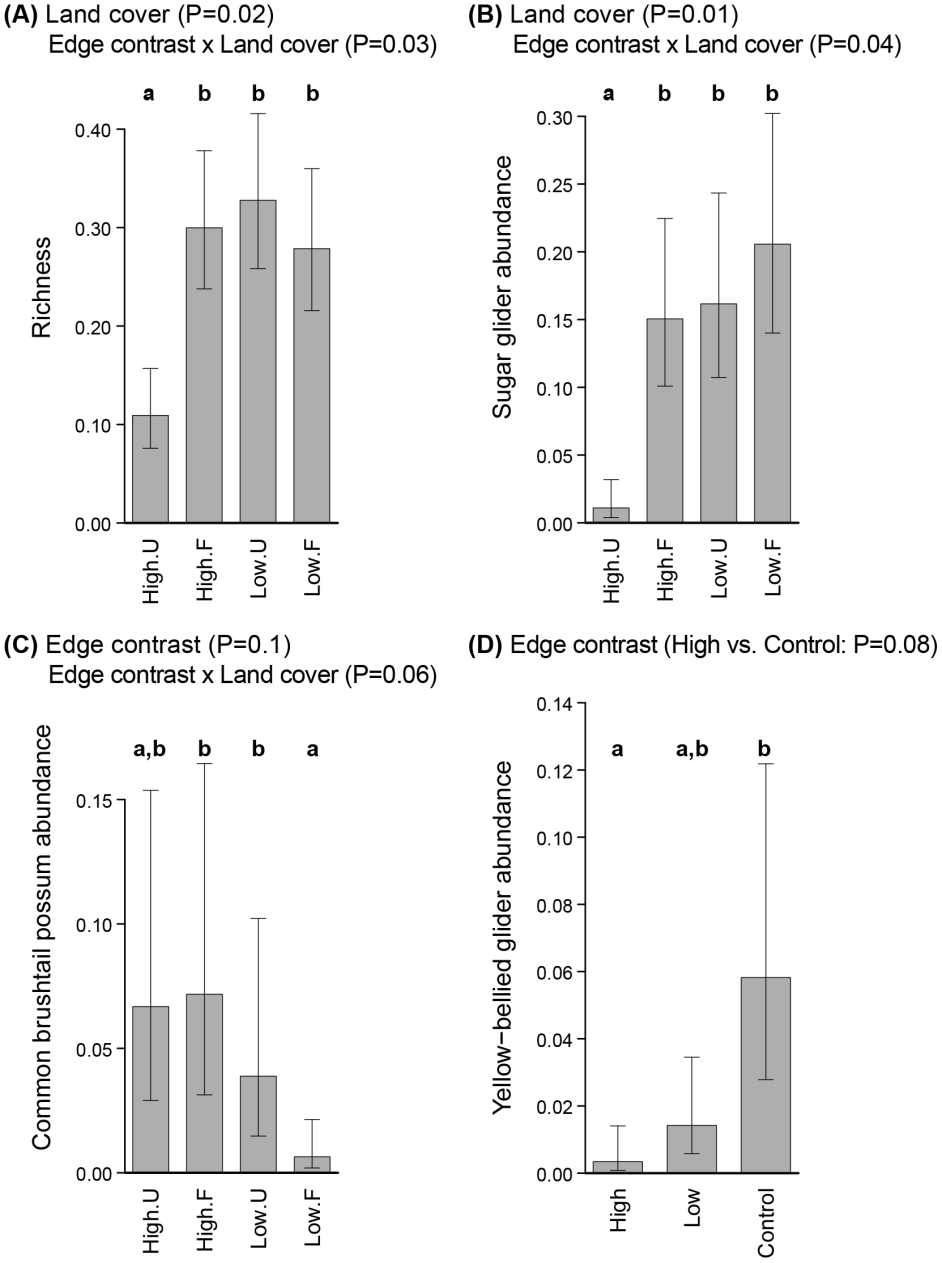
Predicted mean values of arboreal marsupials per 100 m spotlight transect from Generalized Linear Mixed Models (best models). (A) Species richness, (B) sugar glider abundance, (C) common brushtail possum abundance, and (D) yellow-bellied glider abundance. Codes of edge contrasts: High = high housing density; Low = low housing density; Control. Codes of land cover: F = forest; U = urban. Bars indicate standard error. Estimated values were predicted for a single spotlighting pass. Significant *P*-values of the relevant variables in the GLMMs and significant contrast tests are shown on the top of each graph. Different letters on the top of each bar indicates significant differences of contrast tests at a 90% confidence level.

From data on the six species recorded, we did not find any effect of distance to the urban boundary on species richness (Wald contrast tests and Wald test, *P*>0.1). Richness was lowest in urban cover with high housing density (*P*<0.08) (Figure 4A, Figure S1A).

### Responses by Individual Species

We found that common ringtail possum abundance increased with urban cover (*P* = 0.08), but it exhibited a steep reduction in abundance toward the interior of the urban cover with high housing density (*P* = 0.08) (Figure 3B).

There was no significant effect of distance to the urban boundary on the abundance of the sugar glider, common brushtail possum or yellow-bellied glider. Sugar glider abundance was lowest in urban cover with high housing density (*P*<0.07) (Figure 4B, Figure S1B). In contrast, common brushtail possum abundance was lower in forest adjacent to low-housing density developments when compared to both the adjacent urban cover (*P* = 0.04), and the forest adjacent to high-housing density developments (*P*<0.1) (Figure 4C, Figure S1C). Yellow-bellied glider abundance was best described by edge contrast, decreasing in abundance at hard edges (i.e. urban cover with high housing density and adjacent forest) when compared with forest controls (*P* = 0.08) (Figure 4D). This was the only species where the backward selection procedure discarded both distance to the urban boundary and land cover as relevant predictors; incorporating forest controls.

### Spatial Autocorrelation

We did not find evidence of spatial autocorrelation in models’ residuals between sites. The residual semivariance did not increase with distance between sites. Further, Moran’s I autocorrelation indices were not significant (*P*>0.05; Table S2).

### Differences in Vegetation Structure Among Transects

**Figure 5:**
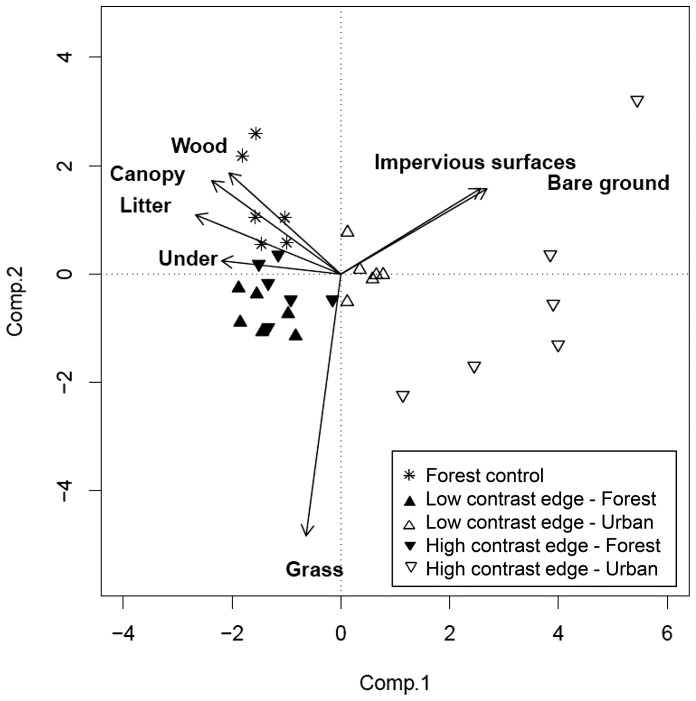
Component loading of principal component analysis (PCA) of vegetation variables on transects surveyed in south-eastern Australia. Under: understory; Wood: woody debris.

The first three components of the PCA explained 56%, 20% and 10% of variation in vegetation structure on spotlight transects, respectively (Table S3A). Component 1 was positively correlated with the proportion of bare ground and impervious surfaces, and negatively correlated with litter, woody debris, understory and canopy cover. Therefore, Component 1 represented a gradient of increasing clearing of the vegetation and its replacement with impervious surfaces (Figure 5). Component 2 had a high negative correlation with the proportion of grass, and a positive, but low correlation (≤0.3) with all remaining variables (e.g. canopy, woody debris, impervious surfaces), and thus represented a gradient of reduction in grass cover and an increase of the other structures (Figure 5). Component 3 was positively correlated with understory and negatively correlated with woody debris, representing increasing shrub density and the reduction of woody debris.

We found significant differences among transect classes for the first two components (Table S3B). The first component showed that control sites had significantly higher canopy cover, litter, understory and woody debris, and less bare ground and impervious surfaces than both low-density housing sites (*P* = 0.07) and high-density housing sites (*P* = 0.0001); and high-density housing sites had significantly more bare ground and impervious surfaces than low-density housing sites (*P* = 0.01) (Figure 5). Forest cover was characterized by a high proportion of canopy cover, understory, litter and woody debris, whereas the urban cover was characterized by the high proportion of bare ground and impervious surfaces. The difference between forest and urban cover was always significant within the same housing density (*P*<0.0002) and among all housing densities (*P*<0.04). The second component showed that both high and low-density housing sites had significantly a higher proportion of grass than forested controls (*P* = 0.006). As a result of the high variance in vegetation related to the transect classes (i.e. both housing density and land cover), we did not use the main components for fitting additional GLMMs to avoid overparameterizing models with redundant variables.

## Discussion

Urbanization is a key process threatening biodiversity worldwide. In our empirical work on arboreal marsupials, we did not find the expected gradual change in species abundance across forest-urban edges based on simple habitat characteristics of the adjacent patches. Rather, we identified a broad range of responses suggesting that considering habitat characteristics in isolation lacks predictive power. Therefore, we present a model predicting the trajectory of animal responses across edges based on three fundamental steps:

1. the habitat quality/habitat preference between juxtaposed patches,
2. the species response with the proximity to the adjacent habitat, and
3. the extent of the spillover/sensitivity to adjacent habitat boundaries.

In the following section we discuss the responses of each arboreal marsupial, followed by a predictive framework of edge effects.

### Arboreal Marsupials Across Urban-forest Edges

Species loss and biotic homogenization have been proposed as one of the main impacts of urbanization on biodiversity [43], [44]. This decline in vertebrate richness in urban landscapes has been associated with an increased human and building density [45]–[47], and the reduction of native vegetation such as canopy cover [46], [48]. In our study, species richness was lowest in high-density housing developments, but there was no significant impact on the adjacent forest. Therefore, the significant reduction of the vegetation in high-density housing developments (and canopy cover in particular) appears likely to be the main cause of decline of arboreal marsupials in urban environments.

The total abundance of arboreal marsupials increased towards the interior of low-density housing developments, whereas the opposite trend was found in high-density housing developments. It has been widely proposed that different edge contrasts produce a change in magnitude or extent of the response but not a change in its direction [10], [11]. In contrast to other studies reporting that urbanization increases the total abundance of a few dominant species [46], [49], our results for arboreal marsupials revealed that both species richness and total abundance declined with higher levels of urbanization (i.e. high housing density), and the effect was reversed in low-density housing developments in this environment. Low-density housing developments provided suitable habitat for most of the species, probably as a result of the moderate level of clearing of the vegetation that increased the diversity of niches [50]. Also, the bias towards the development of private lands on higher productivity sites in the study area [51] might have contributed to the increased abundance of arboreal marsupials in low-density housing developments.

At the species level, the sugar glider did not respond to distance to the urban boundary. Instead, our result implied habitat loss in high-density housing developments, but no negative effect on adjacent forests, and that low-density housing developments provided a suitable habitat. This species is often found in forest strips and forest fragments [52], consistent with our observations that they had high abundances in low-density housing developments which are more open than forests, but with retained tree cover. The lack of a negative effect on sugar glider abundance in the adjacent forests might be a result of its non-response to the proximity of urban areas. However, their high degree of arboreality, along with the lack of canopy cover in high-density housing developments might have limited their ability to cross the urban boundary, leading to the restricted spillover of individuals from forests to urban areas.

The yellow-bellied glider was less abundant in high-density housing developments and the adjacent forests compared to forested controls, suggesting its sensity to urban development at a large spatial scale. This forest-interior species needs large areas of forest to meet their dietary requirements [52], [53] and is sensitive to the effects of habitat loss and fragmentation [52], [53]. This may explain its avoidance of forest boundaries and its sensitivity to urban disturbance beyond 300 m from the urban boundary. Although edge effects on animals have been studied mainly over short distances (e.g. ≤300 m [11], [19], [30], [54]), an extended edge effect from urban developments has been reported in carnivorous mammals in North America, with both specialist and behaviourally-plastic species responding at several kilometres to the urban boundary [55].

Previous studies have found that the common ringtail possum, as well as other ringtail possums, have higher abundances at edges in forested environments [30], [56], as a result of either an increased foliage density [30] or access to complementary resources [57]. In contrast, we found a neutral response with the proximity to the urban boundary, probably as a result of a lack of immigrants from the high-density housing development [58]; and a reduction in its abundance towards the interior of high-density housing developments. This response trajectory may be the result of a spillover of animals from forest to urban areas; with the strong reduction of the abundance with increasing distance to the forest representing dispersing animals across hard edges.

The degree of specialization, such as arboreality, denning requirements and feeding habitats, as well as dispersal, home range sizes and dependence on primary forests [55], [59] might interact to explain the different response patterns observed. Non-volant species (e.g. possums) might be favoured by urban areas, because they are not strictly arboreal like gliders, and are able to move along the ground [52]. Despite the potential ability of the common ringtail possum to colonize new environments because it is not an obligate cavity-dependent species [60], the common brushtail possum was the most successful species in colonizing high-density housing developments.

Among arboreal marsupials, the common brushtail possum is able to use new resources provided by urban areas (e.g. rubbish, gardens and vegetable patches as food supply; along with roofs and other building structures for denning) [61], [62], indicating it is an “urban adapter”. “Urban adapter” species are native species that increase their abundance in residential areas (i.e. suburbia) [1]. For example, among carnivorous mammals, raccoons (*Procyon lotor*, Linnaeus 1758) in North America are positively associated to residential areas probably as a result of the use of refuse as a food supply [63]. Feeding habits has been proposed as the main mechanism underpinning the abundance of animals in urban areas, with omnivorous species positively related to urbanization whereas specialized species are diminished [43], [54], [63]. In addition, those species with high reproductive potential [64] as well as those which can use buildings as resting or nesting sites [65] will benefit from urban environments.

### A Predictive Model of Edge Effects

Based on habitat characteristics, we expected a gradual change in the abundance of all species across edges, as a result of the spillover of animals from forests to urban areas (Figure 1). However, we found only partial support for this response in the common ringtail possum (Figure 3).

**Figure 6:**
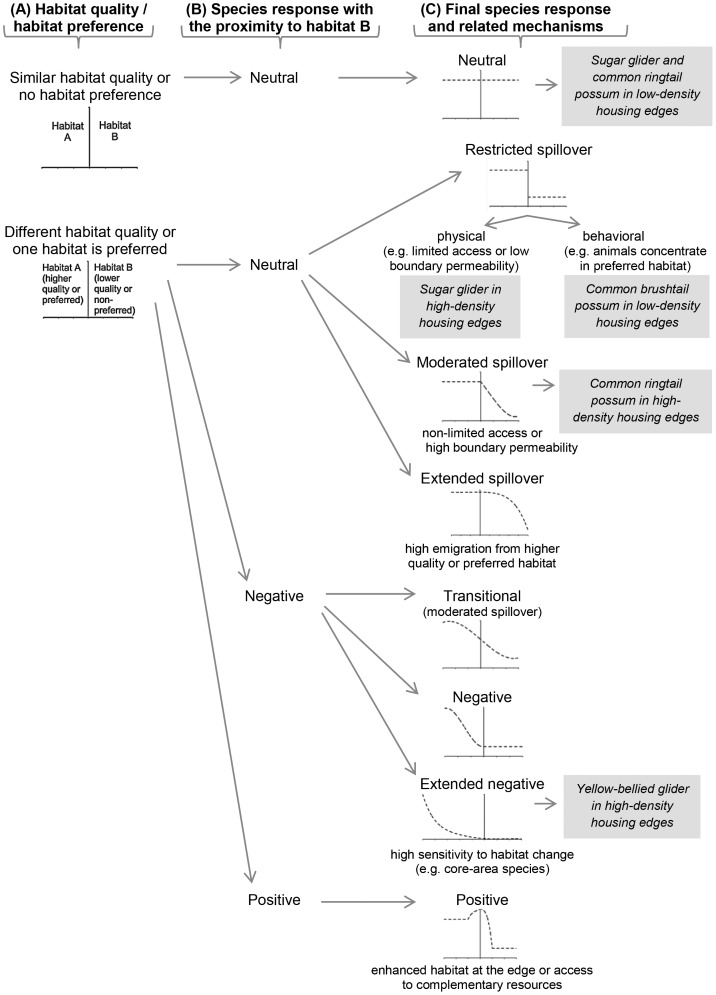
New predictive model of edge effects in animals. Columns indicate three consecutive steps to predict the final animal response. Graphs show the predicted abundance of a species (dashed line) in adjacent habitats. The vertical line in each graph represents the boundary between two habitat patches. (A) The first step in the model proposes both habitat quality and habitat preference defining the initial response between two adjacent habitat patches. (B) The second step identifies three kinds of animal responses according to the proximity of the adjacent habitat. (C) The last step outlines the final response trajectory and related mechanisms modifying the response. Mechanisms modifying the response trajectory were related to species attributes and behavior (e.g. access to adjacent patch, avoidance to emigrate from preferred habitat, sensitivity to habitat change), population dynamics (e.g. density-dependence driving emigration), and patch attributes (e.g. permeability to emigration). Text boxes show the observed responses by each species in different edge contrasts in our study.

Habitat quality is a basic element explaining the distribution of animals (e.g. ideal free distribution) [66]; but to understand the distribution of animals in adjacent habitats, we need to consider more detailed knowledge of both a given species and the environments involved [11]. According to Lidicker [67], there are two fundamental kinds of edge effects present in vertebrates: a matrix effect and an ecotonal effect. A matrix effect is observed when the response of animals across edges is a result of their response in each habitat type in isolation (Figure 6A); whereas an ecotonal effect is observed when animals respond to the proximity of habitat boundaries [67] due to the influence of an adjacent patch [15] (Figure 6B). An ecotonal effect can produce different response trajectories [13]. However, ecotonal effects are commonly classified as positive, negative or neutral if the response increases, decreases or does not change with decreasing distance from the edge, respectively [15], [24], [67] (Figure 6B).

The final trajectory of the animal response across edges is defined by different mechanisms (Figure 6C). For example, the extent of the spillover of individuals on the adjacent habitat and the species sensitivity with the proximity to the adjacent patch will be influenced by the biology and behaviour of the species [19], [67], as well as population dynamics [66] and attributes of the juxtaposed patches, such as boundary permeability to emigration [15], [68], [69]. Therefore, we integrated these variables with our results, to develop a new model of edge effects to help better predict animal responses across edges.

In our model, the initial response is influenced by both habitat quality and habitat preference [15], [19], [24]. When habitat quality or resources are similar between patches or species show no preference for a particular habitat patch, a neutral response is expected at both sides of the boundary (e.g. sugar glider and common ringtail possum in low-density housing developments and adjacent forests) (Figure 6).

When one patch has significantly higher habitat quality or is preferred, animals will reach a higher abundance in that patch when compared to the adjacent patch. As habitat quality or preference differs between patches, animals might respond to the proximity of the adjacent patch. Then, the trajectory of the response may be neutral, negative or positive with decreasing distances to the boundary.

A neutral response with the proximity to the adjacent habitat (i.e. no change in abundance with distance from the preferred-habitat side of the edge) might generate three main kinds of final response trajectories across habitat edges which are influenced by the extent of the spillover (i.e. proportion of animals crossing the habitat boundary): restricted spillover, moderated spillover, and extended spillover. First, a restricted spillover might be a result of either physical or behavioural mechanisms. Physical mechanisms might include limited access to the adjacent patch (e.g. sugar glider spillover is limited from forest to high-density housing developments as a result of its specialized movement that depends on vertical structures that allow gliding), or low boundary permeability [15], [68]. Behavioural mechanisms restricting spillover may involve a reluctance to cross habitat boundaries as a result of increased predation risk [70], or the concentration of individuals in preferred habitats without density-dependent processes driving emigration to the non-preferred patch (e.g. common brushtail possum in low-density housing developments had a limited spillover from the preferred urban habitat into the adjacent forest). Second, a moderated spillover will consist in animals crossing the boundary but only a few colonizing the adjacent habitat (e.g. common ringtail possum spillover from forest to high-density housing developments). Third, an extended spillover will be found if emigration from higher quality or preferred habitat is high [15], [68]. This increased spillover may occur with density-dependent emigration, such as when territorial species saturate optimal habitats and boundary permeability to emigration is high (e.g. in populations with high density, young common brushtail possums searching for territories are frequently subject to conspecific conflicts [71], which might increase their emigration from the overcrowded high-density housing developments to adjacent forest).

A negative response with the proximity to the adjacent habitat can generate three main final response trajectories across habitat edges. A “transitional” [24] response across the edge (or “mutual influence” [15]), allows a moderated spillover of animals into the adjacent habitat. A negative response across the edge (or “negative influence” [15]) results when a species reaches the same abundance at the boundary as in the adjacent habitat patch. An extended negative response is found when the animal abundance declines a long way away from a boundary. An extended negative response will be expected in species sensitive to habitat change, such as core-area species (e.g. yellow-bellied glider in forest adjacent to high-density housing developments) and species of conservation concern [19].

Finally, animals might respond positively to the proximity of habitat boundaries. For example, if resources are concentrated at the edge or different resources can be found at each side of a boundary, animal abundance will increase with the proximity to the habitat boundary [11], [15].

Although our model was primarily based on the trajectory of the animal responses found in our empirical work, a framework considering three basic elements (i.e. habitat quality/habitat preference, species response with proximity to the adjacent habitat, and factors determining spillover extent/sensitivity to habitat boundaries) will provide useful insights when predicting animal response across edges. We believe our framework continues the integration of knowledge on edge effects, encouraging both scientists and managers to develop and test predictions in the field.

### Implications for Conservation and Urban Planning

Our results have important implications for both conservation and urban planning. First, our predictive model of edge effects provides useful insights to guide urban planning. For example, it suggests that forest-dependent species exhibit multiple responses to a modified environment. Consequently, different strategies must be considered to avoid or mitigate impacts on a particular faunal group. Moreover, the effects of one environment on a species living in the adjacent habitat will depend on several factors that include not only attributes of the adjacent patches, but also the ecology, biology and behaviour of the species. Therefore, to appropriately predict and mitigate the impact of urbanization on biodiversity, a detailed understanding is needed of the species and the environment involved.

Second, managers and urban planners must be aware of the negative impacts of high-density housing developments on arboreal marsupials. In contrast, low-density housing developments have allowed the persistence of most arboreal marsupials. These results agree with studies that have found a positive effect of low urban density on native mammals in North America [55]. Further, low contrast edges have been shown high value in conserving forest marsupials in urban environments elsewhere in Australia [22]. The main structural difference between high and low-density housing developments was the reduction of native vegetation and key habitat structures (e.g. trees) in high-density housing developments, and their replacement by bare ground and impervious surfaces.

Third, although high-density housing developments had no significant impact on most response variables measured in the adjacent forests, there may have been undetected effects. For example, the neutral response to the boundary found on most species inhabiting forests next to urban developments might be a result of young individuals being displaced close to the forest boundary by adults [22]. Further, high-housing density developments had a negative impact on the abundance of yellow-bellied glider in the adjacent forest, the only threatened species recorded in the study area. As the impact on the yellow-bellied glider extended beyond 300 m from the urban boundary, high-density housing developments must be several hundred meters away from conservation areas (e.g. national parks and reserves) to avoid reducing forest core area for this species. However, at the planning stage of future urban developments, including buffer zones larger than 300 m into projected urban areas might be counter-productive for conservation purposes, as larger forested areas will be released and be subject to land use change. If the negative effects of high-housing density developments are not reversed, they will pose an increased threat to most species of the arboreal marsupials not only through habitat loss, but also by having an extended impact on sensitive species living in adjacent forests.

Finally, the overall impact of low versus high-density housing developments remains unclear [72]. While Sushinsky et al. [73] state that the impacts of urban development on bird distributions may be reduced with an increased housing density, our results on arboreal marsupials demonstrated the opposite trend. We suggest that future research must be focused on: (1) improving land planning by comparing the overall impacts of different styles and configurations of urban development; and (2) developing management strategies to mitigate the current impacts of high-housing density developments. For example, Fontana et al. [47] found that variables subject to management, such as canopy cover, have a greater effect on bird assemblages than human population density; and Palomino & Carrascal [46] conclude that the negative effects of urbanization on forest birds may be reversed if large mature tree cover is provided. When strategies are compared, the retention of original native vegetation may be more cost-effective than vegetation restoration in conserving biodiversity [1]. New research should quantify the effect of both increasing vegetation and retaining the structural complexity of the natural vegetation, in mitigating the impact of high-density housing developments on forest-dependent species.

## Conclusions

Our study provides new understanding of animal responses across urban-forest edges on a large spatial scale and offers useful insights to guide urban planning. We argue that habitat characteristics are among the multiple factors influencing the animal response across habitat edges. To accurately predict animal responses across edges, and inform urban planning, factors that need to be considered are: (1) the habitat quality/habitat preference, (2) the species response with the proximity to the adjacent habitat, and (3) the extent of the spillover/sensitivity to habitat boundaries. We found that high-density housing developments had negative effects on arboreal marsupials, whereas low-density housing developments provided suitable habitat for most of the arboreal marsupials. As a result of the broad range of species responses, we propose two fundamental strategies to minimize the impacts of urban developments: (1) reduce the loss of forest core area at the planning stage, to limit impacts on sensitive species; and (2) mitigate the environmental impact of high-density housing developments on forest-dwelling species by providing key habitat structures that may facilitate the movement of animals and promote colonization of urban environments.

## Supporting Information

Figure S1 **Differences in mean levels of multiple comparison tests.** (A) Species richness, (B) sugar glider abundance, and (C) common brushtail possum abundance in log scale of multiple comparisons of edge contrast and land cover combinations. Codes of edge contrasts: High = high housing density; Low = low housing density. Codes of land cover: F = forest; U = urban. Bars represent 90% confidence intervals. When confidence intervals do not overlap zero, means between comparison levels are different at a significance level of 0.1.
(TIF)

Table S1 **Number of individuals recorded of each species.**
(DOCX)

Table S2 **Moran’s I autocorrelation index on model residuals.**
(DOCX)

Table S3 **PCA and ANOVA of vegetation variables.**
(DOCX)
