# Supplementary figures and images for: Urbanization Impacts on Mammals across Urban-Forest Edges and a Predictive Model of Edge Effects
Source: PLoS One. 2014 May 8;9(5):e97036. doi: 10.1371/journal.pone.0097036 (PMC4014578; doi:10.1371/journal.pone.0097036)

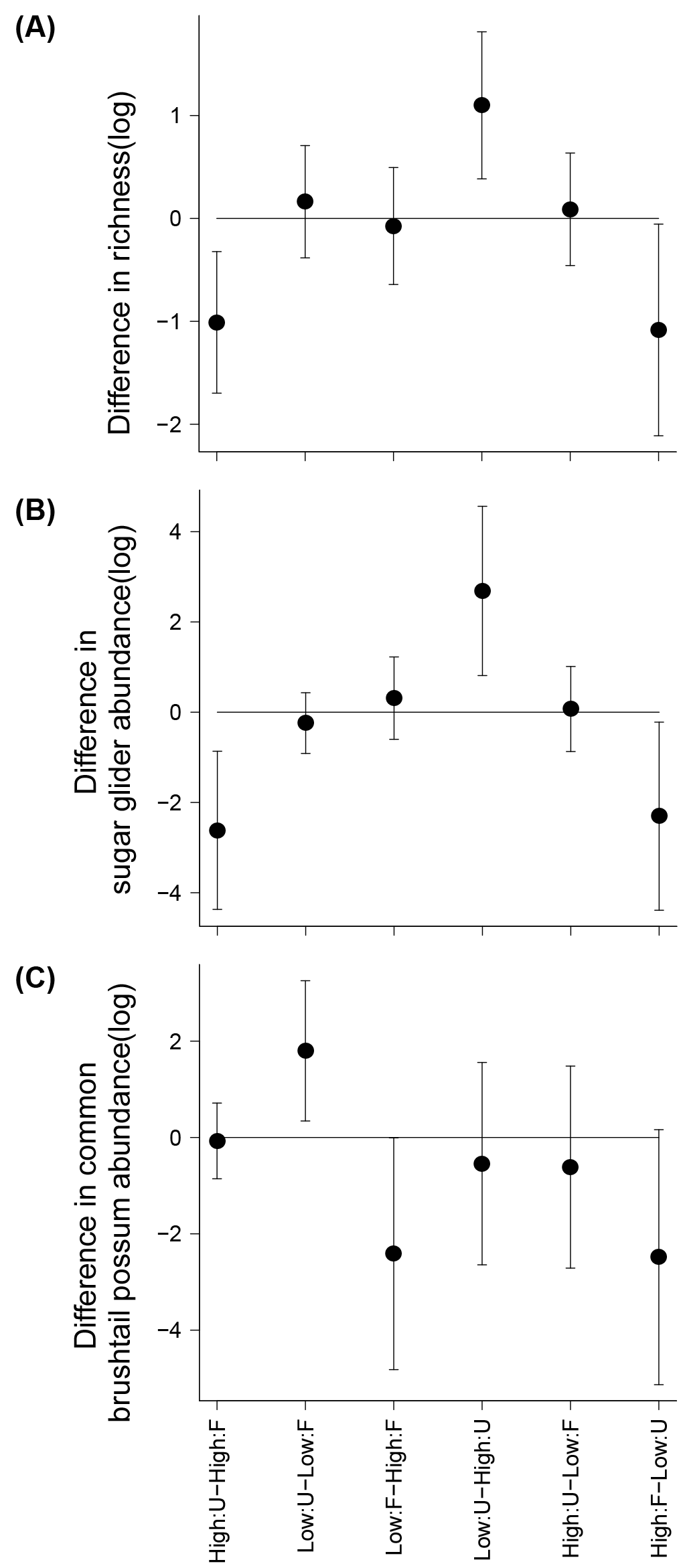

Supplement: Figure S1 — Differences in mean levels of multiple comparison tests. (A) Species richness, (B) sugar glider abundance, and (C) common brushtail possum abundance in log scale of multiple comparisons of edge contrast and land cover combinations. Codes of edge contrasts: High = high housing density; Low = low housing density. Codes of land cover: F = forest; U = urban. Bars represent 90% confidence intervals. When confidence intervals do not overlap zero, means between comparison levels are different at a significance level of 0.1. (TIF) [file pone.0097036.s001.tif]
