# Supplementary material for: Urbanization Impacts on Mammals across Urban-Forest Edges and a Predictive Model of Edge Effects
Source: PLoS One. 2014 May 8;9(5):e97036. doi: 10.1371/journal.pone.0097036 (PMC4014578; doi:10.1371/journal.pone.0097036)
Supplement: Table S1 — Number of individuals recorded of each species. (DOCX) [file pone.0097036.s002.docx]

Table S1. Number of individuals recorded of each species by edge contrast and land cover in south-eastern Australia.

| Species | Edge contrast: | Forest control | Low | | High | |
| --- | --- | --- | --- | --- | --- | --- |
|  | Land cover: |  | Forest | Urban | Forest | Urban |
| Common brushtail possum | |  |  |  |  |  |
| *Trichosurus vulpecula* | | 1 | 2 | 13 | 19 | 24 |
| Common ringtail possum | |  |  |  |  |  |
| *Pseudocheirus peregrinus* | | 6 | 8 | 12 | 2 | 5 |
| Sugar glider | |  |  |  |  |  |
| *Petaurus breviceps* | | 14 | 14 | 11 | 13 | 1 |
| Yellow-bellied glider | |  |  |  |  |  |
| *Petaurus australis* | | 11 | 2 | 1 | 1 | 0 |
| Greater glider | |  |  |  |  |  |
| *Petauroides volans* | | 0 | 0 | 0 | 3 | 0 |
| Feathertail glider | |  |  |  |  |  |
| *Acrobates pygmaeus* | | 0 | 0 | 0 | 1 | 0 |
| Total | | 32 | 26 | 37 | 39 | 30 |
