# Supplementary material for: Urbanization Impacts on Mammals across Urban-Forest Edges and a Predictive Model of Edge Effects
Source: PLoS One. 2014 May 8;9(5):e97036. doi: 10.1371/journal.pone.0097036 (PMC4014578; doi:10.1371/journal.pone.0097036)
Supplement: Table S2 — Moran’s I autocorrelation index on model residuals. (DOCX) [file pone.0097036.s003.docx]

Table S2. Statistics of Moran’s I autocorrelation index on model residuals.

| Model | Observed | Expected | sd | *P* |
| --- | --- | --- | --- | --- |
| Total abundance | -0.0069 | -0.0141 | 0.016 | 0.66 |
| Species richness | -0.0266 | -0.0141 | 0.016 | 0.44 |
| Common brushtail possum abundance | -0.0045 | -0.0141 | 0.016 | 0.54 |
| Common ringtail possum abundance | 0.0135 | -0.0141 | 0.015 | 0.07 |
| Sugar glider abundance | -0.0201 | -0.0141 | 0.016 | 0.70 |
| Yellow-bellied glider abundance | -0.0044 | -0.0112 | 0.014 | 0.61 |
