# Supplementary material for: Urbanization Impacts on Mammals across Urban-Forest Edges and a Predictive Model of Edge Effects
Source: PLoS One. 2014 May 8;9(5):e97036. doi: 10.1371/journal.pone.0097036 (PMC4014578; doi:10.1371/journal.pone.0097036)
Supplement: Table S3 — PCA and ANOVA of vegetation variables. (DOCX) [file pone.0097036.s004.docx]

Table S3. (A) Summary of the Principal Component Analysis (PCA) of vegetation variables on transects surveyed in south-eastern Australia. Bold denotes factor loadings >0.35. (B) Two-way ANOVA on the first three components from Principal component analysis (PCA) of vegetation variables. Bold denotes *P*<0.1.

| **(A)** | | | | | |
| --- | --- | --- | --- | --- | --- |
|  | | Component loadings | | | |
| Site structure variables | Range (mean) | | C. 1 | C. 2 | C. 3 |
| Bare ground | 0 - 0.18 (0.06) | | **0.447** | 0.262 | 0.192 |
| Litter | 0 - 1 (0.80) | | **-0.445** | 0.182 | 0.176 |
| Grass | 0.35 - 0.96 (0.74) | | -0.107 | **-0.805** |  |
| Impervious surface | 0 - 0.31 (0.028) | | **0.429** | 0.263 | 0.128 |
| Woody debris | 0 - 0.21 (0.062) | | -0.343 | 0.311 | **-0.632** |
| Understory | 0 - 0.78 ( 0.33) | | **-0.365** |  | **0.716** |
| Canopy | 0 - 0.93 ( 0.56) | | **-0.395** | 0.287 |  |
| Proportion of variance explained | | | 0.562 | 0.195 | 0.098 |
| Cumulative proportion of variance explained | | | 0.562 | 0.758 | 0.857 |

| **(B)** | | | | | |
| --- | --- | --- | --- | --- | --- |
| Source of variation | *DF* | *SS* | *MS* | *F* | *P* |
| Component 1^a^ |  |  |  |  |  |
| **-edge contrast** | **2** | **8.52** | **4.26** | **13.14** | **<0.001** |
| **-land cover** | **1** | **19.30** | **19.30** | **59.53** | **<0.001** |
| -edge contrast x land cover | 1 | 0.01 | 0.01 | 0.03 | 0.87 |
| -residuals | 25 | 8.10 | 0.32 |  |  |
| Component 2 |  |  |  |  |  |
| **-edge contrast** | **2** | **13.36** | **6.68** | **6.46** | **0.006** |
| -land cover | 1 | 0.68 | 0.68 | 0.66 | 0.43 |
| -edge contrast x land cover | 1 | 1.12 | 1.12 | 1.15 | 0.29 |
| -residuals | 25 | 25.88 | 1.04 |  |  |
| Component 3 |  |  |  |  |  |
| -edge contrast | 2 | 3.05 | 1.52 | 2.23 | 0.13 |
| -land cover | 1 | 0.20 | 0.20 | 0.29 | 0.60 |
| -edge contrast x land cover | 1 | 0.48 | 0.48 | 0.70 | 0.41 |
| -residuals | 25 | 17.06 | 0.68 |  |  |

^a^ log transformed to meet ANOVA assumptions.
